# Using Routine Data Sources to Feed an Immunization Information System for High-Risk Patients—A Pilot Study

**DOI:** 10.3389/fpubh.2018.00037

**Published:** 2018-02-16

**Authors:** Domenico Martinelli, Francesca Fortunato, Stefania Iannazzo, Maria Giovanna Cappelli, Rosa Prato

**Affiliations:** ^1^Department of Medical and Surgical Sciences, University of Foggia, Foggia, Italy; ^2^Directorate-General of Health Prevention, Ministry of Health, Rome, Italy

**Keywords:** chronic illness, high-risk patients, underlying medical conditions, comorbid disorders, vaccination, immunization information system, data-linkage

## Abstract

**Background:**

Vaccine-preventable diseases among high-risk patients are a public health priority in high-income countries. Most national immunization programs have included vaccination recommendations for these population groups but they remain hard-to-reach and coverage data are poorly available. In a pilot study, we developed and tested an automated approach for identifying individuals with underlying medical conditions to feed an immunization information system (IIS).

**Methods:**

We reviewed published recommendations on medical conditions that indicate vaccination against influenza, pneumococcal disease, meningococcal disease, hepatitis A, and hepatitis B. For each medical condition, we identified the International Classification of Diseases, Ninth Revision, Clinical Modification diagnosis and procedure codes, the user fee exempt codes and the Anatomical Therapeutic Chemical Classification System codes and we reported these data in correspondence tables. Using these tables, we extracted three lists of patients recorded in three current data sources between 2001 and 2010 in the Apulia region of Italy: the hospital discharge registry, the user fee exempt registry, and the drug prescription registry. Using a unique personal identification number, we linked these three lists of patients with the regional IIS (2012 database), obtaining a list of patients with chronic diseases eligible for vaccination. We tested completeness, sensitivity, and positive predictive value (PPV) of this approach by asking a sample of 28 general practitioners (GPs) to evaluate the matching between a sublist of patients with clinical recommendations for influenza vaccination and the GPs individual subjects medical records.

**Results:**

We included a total of 1,204,496 subjects with underlying medical conditions eligible to receive any of the aforementioned vaccinations. Of these, 9% were identified in all three data sources, 18% in two sources, and 73% in one source. The completeness of this automated process in identifying GPs high-risk patients eligible for influenza vaccination was 88.9% [95% confidence intervals (95% CI): 88.1–89.8%], with a sensitivity of 69.2% (95% CI: 67.7–70.6%) and a PPV of 85.7% (95% CI: 84.4–86.8%).

**Conclusion:**

The high completeness of the methodology used for identifying high-risk patients in current data sources encouraged us to apply this approach for feeding the regional IIS.

## Introduction

In the past few decades, the availability of a growing number of new vaccines and their inclusion in immunization programs has provided the opportunity to cover the whole life course. On the other hand, the higher survival rate of people with chronic and immunocompromising conditions has increased the demand for specialist advices on vaccine indications and contra-indications (1).

Despite several high-income countries have included vaccination recommendations for subjects with chronic diseases in their national immunization schedules (2, 3), these population groups remain extremely hard-to-reach and vaccine coverage data are poorly available.

The national seasonal influenza vaccination survey conducted in December 2015 by the European Centre for Disease Prevention and Control (ECDC) showed that all Member States of the European Union recommended influenza vaccination for people with immunosuppression due to diseases or treatment, metabolic disorders, chronic pulmonary, cardiovascular and renal diseases, but the vaccination coverage data were provided only by seven States and ranged from 21 to 71.8% (4).

A specific tool for data collection in those subjects with chronic medical conditions is used in England where the “ImmForm survey” makes monthly available provisional data of seasonal influenza vaccine uptake among general practitioners (GPs) patients (5). Most commonly, vaccination coverage in adults at increased risk is estimated by utilizing data from nationally representative surveys such as, in the United States, the Behavioral Risk Factor Surveillance System and the National Health Interview Survey (6, 7), and, in Europe, the “Gaining Health” strategy (8). In addition, a significant number of studies reporting data on vaccination in subjects suffering from chronic diseases have been published (9–12).

Immunization information systems (IIS) are defined as confidential, population-based, computerized databases that record all immunization doses administered by participating providers to persons residing within a given geopolitical area (13). IIS have great potential to be the most robust and systematic approach to providing better data on when, where, and who received which vaccine and to inform clinicians, patients, citizens, and public health authorities on the key components of any immunization program (1).

Immunization information system can help immunization programs identify populations at high risk for vaccine-preventable diseases (13); they form a fundamental platform either when data are collected within the same information system as morbidity data or, most importantly, by easily linking individual-level vaccination records with other medical records and health outcome databases (14). Since chronically ill patients need to be in contact with multiple medical primary and specialist health services for continuing care and disease management (15), they leave “trails” in several healthcare and administrative databases. Integration of certain information included in these data systems within the IIS can bring additional strengths in terms of studying comprehensively the impact of vaccines—both effectiveness and safety—as well as in formulating the best possible vaccination programs for high-risk populations (14). Given these features and their potential, IIS may be considered the most useful tool to improve vaccination coverage also among people with chronic illnesses.

In Italy, immunization programs are managed within the National Health Service in the framework of the health systems’ fundamental principles and goals. The Ministry of Health issues the National Immunization Prevention Plan which defines the immunization standards all regions should comply with and sets specific objectives to be reached at the national and regional level in terms of target coverage rates, IIS, infectious diseases surveillance, quality and safety of immunization programs (16–18).

With regard to high-risk subjects, the National Immunization Prevention Plan lists the immunization programs, by vaccine and high-risk subgroup (17, 18), but a major challenge faced in their implementation include the need for an IIS to identify at-risk patients. This relies not only on accurate and complete numerators and denominator populations from different sources for calculating vaccination coverage but also on ensuring that the data captured in the system is reliable (19).

We conducted a pilot study aimed to develop and test an automated approach for identifying high-risk patients in some current data sources to feed an IIS.

## Materials and Methods

The Apulia region of Italy [approximately 4,000,000 inhabitants (20)] holds an IIS (Gestione Informatizzata Anagrafe Vaccinale, or GIAVA) since 2005.

In addition, the following data sources are currently available to produce lists of high-risk patients:

✓ Hospital discharge registry (HDR), which collects data on discharge diagnoses (one main and up to five secondary diagnoses) and procedures of all patients admitted to hospitals in Italy, coded using the International Classification of Diseases, Ninth Revision, Clinical Modification (ICD9-CM).
✓ User fee exempts registry (UFER), in which information on chronic patients entitled to fee exemption for medical consultations and drugs due to their specific medical condition are recorded; in the UFER, each condition is identified by a specific and unique code at regional and national level (21).
✓ Drugs prescription registry (DPR), in which information on drugs prescribed to patients by the health services are stored. Drugs are coded according to the Anatomical Therapeutic Chemical Classification System (ATC).

For the purpose of this pilot study, we reviewed published recommendations on medical conditions that indicate vaccination against influenza, pneumococcal disease, meningococcal disease, hepatitis A, and hepatitis B. For each medical condition, we identified the ICD9-CM diagnosis and procedure codes and the UFER codes, and traced the updated therapeutic protocols within the most commonly used GP practice software for patient management. Thus, we prepared a list of recommended drugs with the respective ATC code, considering only drugs unequivocally ascribable to that medical condition (i.e., insulin for diabetes). The lists of selected ICD9-CM, UFER, and ATC codes, with the adequate references, were reported in different correspondence tables (Data Sheet S1 in Supplementary Material).

Using these tables, we extracted three lists of patients in all age groups with underlying medical conditions recorded in the HDR, the UFER, and the DPR between 2001 and 2010. Then, we linked these three lists of patients with the individual immunization records through the regional IIS (2012 database), using a unique personal identification number. Thus, we obtained a list of patients with chronic diseases eligible for vaccination [chronic patients list (CPL)].

The contribution of each data source (HDR, UFER, and DPR) to the CPL was estimated by dividing the number of subjects extracted from each source by the total number of patients with chronic diseases eligible for vaccination in the CPL.

To test the capacity of this approach for identifying high-risk patients, we invited 28 GPs throughout the region to evaluate the matching between a CPL of children and adults aged ≤65 years with clinical indications for influenza vaccination (CPL_flu_ sublist) and the GPs individual subjects medical records. To assess the completeness of the tool, we asked physicians to identify subjects with chronic illnesses who were vaccinated during the prior 2011–2012 flu season and who were not present in the CPL_flu_. Completeness was estimated by dividing the number of patients included in the CPL_flu_ by the total number of GPs patients with chronic diseases eligible for flu vaccination, together with 95% confidence intervals (95% CI).

Moreover, a two-source capture–recapture method (22) was used to provide an estimate of the total number of patients eligible for flu vaccination (*N*) and who were not present in both CPL_flu_ and GPs individual subjects medical records:

$$N=\frac{\left(N_{\text{A}}+1)\times (N_{\text{B}}+1\right)}{N_{\text{AB}}+1}-1$$

where *N*_A_ was the number of patients in the CPL_flu_, *N*_B_ was the number of GPs patients, and *N*_AB_ was the number of patients common to both sources. CPL_flu_ sensitivity, specificity, and positive predictive value (PPV) and negative predictive value (NPV) together with 95% CI were also estimated.

Analyses were performed in STATA software (version 14; StataCorp, College Station, TX, USA).

### Ethics

The study was conducted according to the principles expressed in the Declaration of Helsinki. The study protocol was approved by the Institutional Review Board at the Apulian Regional Observatory for Epidemiology (PROT: 201/OER/2010, November 19, 2010). Informed consent was not obtained from participants because data from all utilized sources were provided and analyzed anonymously. No identifiable human data were used for this study. The dataset used in this study is not openly available. Data are available from Apulia regional Health Authorities Institutional Data Access for researchers who meet the criteria for access to confidential data.

## Results

As at 2012, a total of 1,204,496 subjects with underlying medical conditions eligible to receive influenza, pneumococcal, meningococcal, hepatitis A, and hepatitis B vaccines were included in the CPL. Of these, 35.8% were in the age group 55–74 years, 51.8% female (Table 1).

**Table 1:** Characteristics of subjects included in the chronic patients list (*N* = 1,204,496) in the Apulia region of Italy, within 2001–2010.

Sex		*N*	%
	Male	580,911	48.2
	Female	623,585	51.8

**Age group**			

	<15 years	80,152	6.7
	15–24 years	52,910	4.4
	25–34 years	68,109	5.6
	35–44 years	112,184	9.3
	45–54 years	170,195	14.1
	55–64 years	244,118	20.3
	65–74 years	186,206	15.5
	75–84 years	184,846	15.3
	≥85 years	105,776	8.8

**Figure 1:**
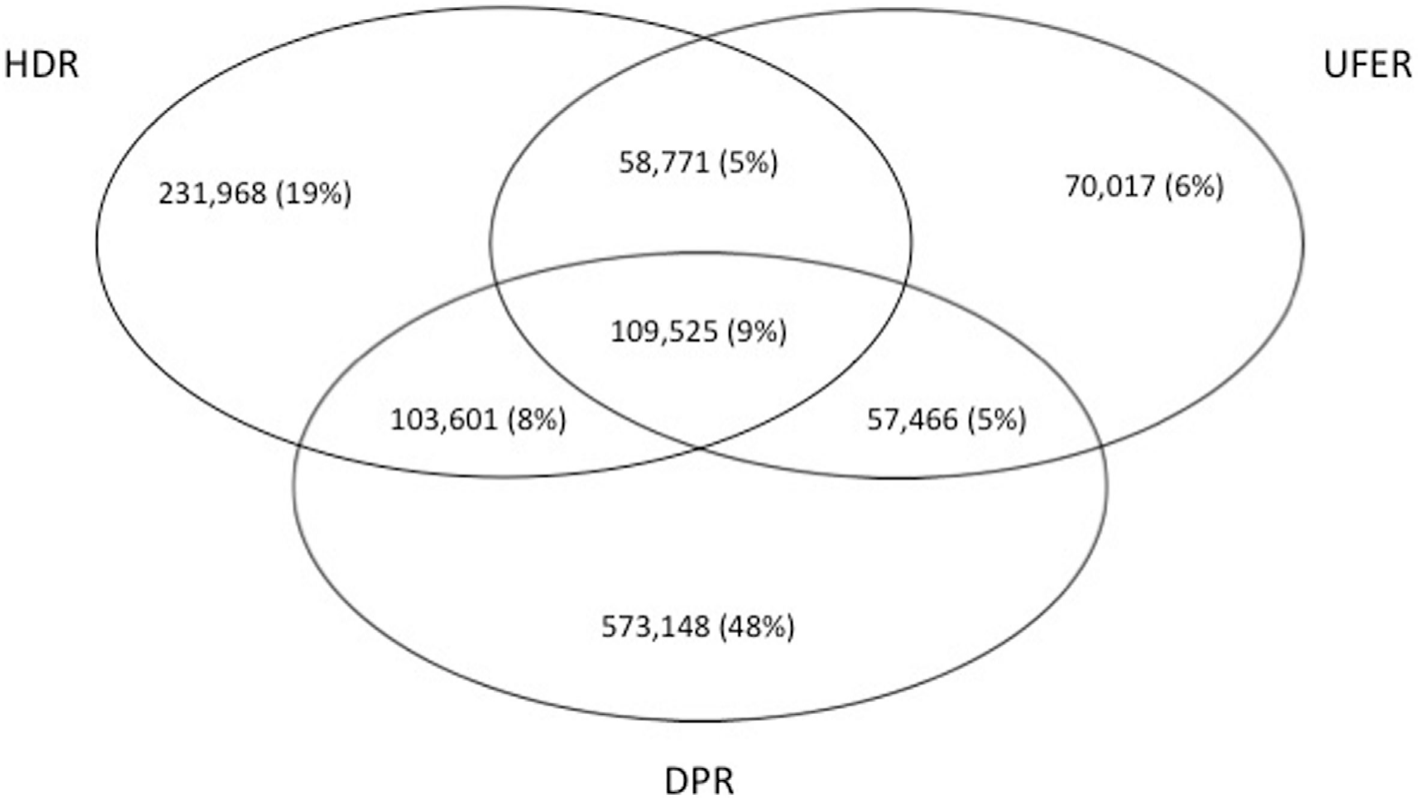
Venn diagram of the contribution of the three sources hospital discharge registry (HDR), user fee exempts registry (UFER), and drugs prescription registry (DPR) to the chronic patients list in the Apulia region of Italy, within 2001–2010.

Each data source examined (HDR, UFER, and DPR) contributed differently to create the CPL: 9% of patients were identified in all three sources, 18% in two sources (HDR and UFER; or HDR and DPR; or UFER and DPR), and 73% in one source (only HDR; only UFER; only DPR) (Figure 1).

A total of 4,316 children and adults aged ≤65 years were included in the CPL_flu_. Of these, 4,023 (93.2%; 95% CI: 92.4–93.9%) patients were matches with the 28 GPs individual subjects medical records; the remaining 293 (7%) were not traced because they died or left the GP’s practice during the examined period. For 2,782 patients (69.1%; 95% CI: 67.7–70.6%), both CPL_flu_ and GPs medical records reported at least one medical indication for flu vaccination.

A total of 536 patients who were vaccinated during the 2011–2012 influenza season were not present in the CPL_flu_. Of these, 466 (86.9%) suffered from mild asthma, dyslipidemia, or hypertension. The completeness of the CPL_flu_ in identifying subjects with medical indications for influenza vaccination was 88.9% (95% CI: 88.1–89.8%).

The two-source capture–recapture method estimated that there were 208 additional patients not captured in both CPL_flu_ and GPs medical records, bringing the total number of patients eligible for flu vaccination to 4,697. The CPL_flu_ sensitivity was 69.2% (95% CI: 67.7–70.6%), the specificity was 30.9% (95% CI: 27.4–34.5%), PPV and NPV were 85.7% (95% CI: 84.4–86.8%) and 14.4% (95% CI: 12.6–16.3%), respectively.

## Discussion

Most high-income countries have reached the point where better data on subjects with chronic medical conditions are essential for ensuring high standard of care to these patients (23). Even if efforts to promote immunization of high-risk subjects are specific objectives of most national immunization plans, many countries report difficulties in estimating numerator and denominator data relating to the numbers of individuals with chronic medical conditions. This reflects a lack of information systems (electronic data sources, such as disease registries) or other standardized methodologies for making these data available (1, 4). Administrative health-care data, collected for managerial reasons, have been extensively used to estimate chronic disease prevalence for the purpose of surveillance. Case finding and chronic disease case ascertainment algorithms are tailored to the structure and type of information that is captured in the specific administrative database (24).

This pilot study has tested the feasibility of a sufficiently easy tool for identifying high-risk patients in current data sources to be linked to an IIS in Italy. By linking three current data sources (HDR, user fee exempt registry, and drug prescription registry) with the regional IIS, we obtained a list of patients with chronic diseases eligible for vaccination. The completeness of this automated process in identifying GPs high-risk patients eligible for influenza vaccination was 88.9% (95% CI: 88.1–89.8%), with a sensitivity of 69.2% (95% CI: 67.7–70.6%) and a PPV of 85.7% (95% CI: 84.4–86.8%).

In the Apulia region of Italy, more than 1,200,000 subjects resulted being hospitalized, having had user fee exemption, or having received medications for chronic conditions and were eligible to receive vaccinations; approximately half of these subjects were adults of retirement age 55–74 years (20). Data from the 2012 National Health Interview Survey showed that approximately half (117 million) of US adults had at least 1 and 1 in 4 adults had at least 2 of the 10 chronic conditions examined (25). In Europe, in 2012, an estimated 52 million EU citizens aged 55–74 reported having a long-standing illness or health problem; this is about half of all people in this age group (26). These figures have important implications in terms of challenges encountered in safeguarding individuals over their lifetime through planning and implementing adequate immunization programs.

In our study, 1 in 4 subjects in all age groups and 1 in 2 adults aged 55–74 years have left “trails” in health care and administrative databases (Table 1). Of the three data sources used to trace patients with chronic illnesses, the DPR made the most meaningful contribution, capturing 48% of cases not identified within the HDR or the UFER (Figure 1). Other studies have suggested that drug prescription registries could represent a gold standard for capturing patients with diabetes (27, 28).

Our CPL approach was not able to identify all patients with underlying medical conditions resided in the study region, as the 90% completeness and the 70% sensitivity of the CPL_flu_ in identifying GPs patients with medical indication for influenza vaccination showed. A study conducted in Italy in 2010 showed that for ischemic heart disease administrative and GP data sources were fairly consistent, for heart failure administrative estimates were consistently higher than GPs’ estimates, while for COPD the prevalence estimates from GP data were consistently higher than the corresponding estimates from the other routine sources (HDR and DPR) (24). These differences may be due to the architecture and type of information that are recorded in the specific administrative database but also to the proportion of patients who have mild or well-controlled diseases (29). In this case, patients have never had either a hospital admission, or a prescription for drugs, or have not received an exemption, therefore escaping our CPL system for identifying them in administrative databases. In our experience, this is a limitation that would be easily overcome making IIS directly linked to GPs patient files.

Other limitations of this study include the secondary use of some existing health care and administrative data sources: (i) using data from hospital discharge database is known to have limitations, such as sensitivity and specificity of coding, differences in coding habits over space and mainly time; (ii) for drug prescription registry the assumption of a 100% case detection could not be verified when drug utilization with no indication is used as a source of case ascertainment (24). Finally, we performed an IIS-feeding approach in a single region with a long-lasting history of experience of healthcare and administrative databases management. Therefore, it may not be directly generalizable to other settings.

This was a first pilot study to test an automated process for the extraction of patients with underlying medical conditions potentially eligible for vaccination from certain routine data sources. As such, it needs to be extensively and properly validated as a unique source for this aim.

Despite these limitations, the highly predictive model we used has showed, as main strength, the ability of capturing a large part of populations at higher risk for vaccine-preventable diseases. Even if it has been initially tested as a “one-shot deal” methodology addressing the need of better numerator and denominator data relating to the numbers of GPs high-risk patients eligible for seasonal flu vaccination, we are applying this approach in real time on data streams moving forward to provide ongoing validation and feedback mechanisms of our IIS. This automated system aims to send direct postal invitations and reminders by letter or tailored text messages or e-mails to people who are due to get vaccinated and automatic reminders to the vaccine provider to call a patient for vaccination (30). Moreover, as part of multi-component interventions to increase vaccination coverage in chronic population, patient engagement, and proactive participation will be enhanced by access to immunization data through devices such as mobile telephones and allowing vaccine recipients to print immunization records (31, 32).

Collecting and integrating more data generate even more data (33). We are planning to integrate other registers (i.e., infectious disease surveillance system, cancer registry, causes of death registry, population-based cancer screening, etc.) in the regional IIS to build a system as optimal as possible.

To the best of our knowledge, this is the first pilot experience in Italy on the use of routine data sources for identifying high-risk patients and feeding a regional IIS. The high completeness of the methodology tested in this study encourages us to pursue further developments.

## Ethics Statements

The study was conducted according to the principles expressed in the Declaration of Helsinki. The study protocol was approved by the Institutional Review Board at the Apulian Regional Observatory for Epidemiology (PROT: 201/OER/2010, November 19, 2010). Informed consent was not obtained from participants because data from all utilized sources were provided and analyzed anonymously. No identifiable human data were used for this study. The dataset used in this study is not openly available. Data are available from Apulia regional Health Authorities Institutional Data Access for researchers who meet the criteria for access to confidential data.

## Author Contributions

DM and RP conceptualized and designed the study, analyzed and interpreted data, and writing the manuscript. FF and MC contributed to the data collection, managed the database, and provided statistical support. SI provided important intellectual input in the various steps of the study. All authors have read and approved the final manuscript.

## Conflict of Interest Statement

The authors declare that the research was conducted in the absence of any commercial or financial relationships that could be construed as a potential conflict of interest.
